# Supplementary material for: Bayesian refinement of protein structures and ensembles against SAXS data using molecular dynamics
Source: PLoS Comput Biol. 2017 Oct 18;13(10):e1005800. doi: 10.1371/journal.pcbi.1005800 (PMC5662244; doi:10.1371/journal.pcbi.1005800)
Supplement: S1 Archive — (BZ2) [file pcbi.1005800.s011.tar.bz2 › gromacs_4.62_bayesian_saxs_refi/share/html/online/pdb.html]

pdb file format


## pdb file format

|  |  |
| --- | --- |
| Main Table of Contents | **VERSION 4.6** |
| GROMACS homepage | **Sat 19 Jan 2013** |

---

### Description

Files with the .pdb extension are molecular
structure files in the protein databank file format. The protein
databank file format describes the positions of atoms in a molecular
structure. Coordinates are read from the ATOM and HETATM records,
until the file ends or an ENDMDL record is encountered.
GROMACS programs can read and write a simlation box in the
CRYST1 entry.
The pdb format can also be used as a trajectory format:
several structures, seperated by ENDMDL, can be read from
or written to one file.

## Example

An pdb file should look like this

```
ATOM      1  H1  LYS     1      14.260   6.590  34.480  1.00  0.00
ATOM      2  H2  LYS     1      13.760   5.000  34.340  1.00  0.00
ATOM      3  N   LYS     1      14.090   5.850  33.800  1.00  0.00
ATOM      4  H3  LYS     1      14.920   5.560  33.270  1.00  0.00
...
...
```

speptide.pdb:
Protein structure file of Ribonuclease A speptide in protein data
bank file format.

Links to descriptions of the pdb format
can be found on the Links page.

---

http://www.gromacs.org
